# Fasting glucose levels at diagnosis and delivery are associated with postpartum glucose abnormalities in GDM women

**DOI:** 10.1007/s00404-025-07953-4

**Published:** 2025-01-28

**Authors:** Ying Gu, Yu Chen, Lingli Hu, Sha Chen, Lin Wang, Mengting Chen, Yanfang Gu, Qi Chen

**Affiliations:** 1 Department of Obstetrics, Wuxi Maternity and Child Health Hospital, Jiangnan University, Wuxi, China; 2 School of Medicine, Nanjing Medical University, Nanjing, China; 3 Wuxi School of Medicine, Jiangnan University, Wuxi, China; 4 Department of Gynaecology, Wuxi Maternity and Child Health Hospital, Jiangnan University, Wuxi, China; 5 Department of Gynaecological Endocrinology, Wuxi Maternity and Child Health Hospital, Jiangnan University, Wuxi, China; 6 Department of Obstetrics and Gynaecology, Faculty of Medical and Health Sciences, The University of Auckland, Auckland, New Zealand

**Keywords:** Gestational diabetes mellitus, OGTT, Fasting glucose levels, Postpartum fasting glucose abnormalities, LGA, Insulin treatment

## Abstract

Women with a history of gestational diabetes mellitus (GDM) significantly increase the risk of developing type 2 diabetes later in life. Although the increased glucose levels typically return to normal range after delivery for most GDM women, a significant proportion of GDM women develop impaired glucose tolerance or overt diabetes after delivery. Several factors associated with postpartum glucose abnormalities have been identified, yet the link between fasting glucose levels at diagnosis of GDM and postpartum glucose abnormalities remains unclear. In this retrospective study with 866 GDM women, we found that 12.5% presented with abnormal postpartum fasting glucose levels (prediabetes). Among those with postpartum fasting glucose abnormalities (n = 109), 63 (57%) women had abnormal fasting glucose levels at diagnosis, indicating an odds ratio of 1.662 (95% CI: 1.12, 2.479, p < 0.001) for these GDM women developing postpartum fasting glucose abnormalities, compared to those GDM women with normal postpartum fasting glucose levels. Additionally, of GDM women with abnormal postpartum glucose levels (n = 109),70 (64%) presented with abnormal fasting glucose levels one day before delivery. The odds ratio for these GDM women presenting with abnormal postpartum fasting glucose levels was 3.751 (95% CI: 2.462, 5.664, p < 0.001) compared to those GDM women with normal postpartum fasting glucose levels. Furthermore, GDM women with additional insulin treatment or delivered an LGA infant significantly increased the risk of developing postpartum fasting glucose abnormalities. Our findings suggest that abnormal fasting glucose levels at diagnosis or shortly before delivery could be a predictive indicator for postpartum glucose abnormalities in GDM women.

## What does this study add to the clinical work

**Table:** 

Abnormal fasting glucose levels at diagnosis or shortly before delivery could be a predictive indicator for postpartum glucose abnormalities in GDM women	
GDM women with additional insulin treatment or delivered an LGA infant significantly increased the risk of developing postpartum fasting glucose abnormalities.	

## Introduction

Gestational diabetes mellitus (GDM) is a common complicated pregnancy, globally affecting approximately 15% of normal pregnancies based on the widely used criteria from the International Association of Diabetes and Pregnancy Study Groups (IADPSG) [1]. GDM is associated with maternal and neonatal adverse outcomes. Additionally, GDM significantly increases the risk of developing type 2 diabetes after 5 to 10 years of childbirth [2]. Therefore, close postpartum glucose monitoring is crucial for women with a history of GDM to detect any glucose abnormalities early and to initiate interventions to prevent or delay the onset of diabetes.

To achieve this, several international guidelines recommended initial glucose testing at 6 to 12 weeks postpartum using either fasting plasma glucose or an oral glucose tolerance test (OGTT) for women with a history of GDM [3, 4]. For most women diagnosed with GDM, the increased glucose levels typically return to normal levels, or diabetes goes away soon after delivery (review in [5]). However, studies have shown that a significant proportion (up to 25%) of women with GDM continued to have impaired glucose tolerance or overt diabetes after delivery (review in [5]). Collectively, these studies suggested the importance of early postpartum care and long-term monitoring in women with a history of GDM [6].

It is well-known that advanced maternal age, obesity, lack of physical activity or sedentary lifestyle change, and non-breastfeeding are associated with an increased risk for postpartum glucose abnormalities and potentially developing type 2 diabetes after 5 to 10 years in women with a history of GDM [7–9]. GDM is internationally defined by one or more blood glucose levels exceeding certain thresholds in a 75 g-OGTT [10]. However, the interpretation can differ due to glucose levels at different time points, which provide information about the body’s response to glucose control.

The association between fasting glucose levels at diagnosis of GDM and postpartum (within 6 weeks) glucose abnormalities remains unclear. Given the substantial rate of progression to diabetes mellitus after GDM, and to identify potential predictive indicators for postpartum glucose abnormalities, we conducted an observational study to explore potential risk factors, such as glucose levels at diagnosis of GDM and adverse pregnancy outcomes, linked to the development of postpartum glucose abnormalities in women with a history of GDM.

## Methods

This study received approval from the Ethics Committee of Wuxi Maternity and Child Health Hospital, China (reference number 2023060921–50). The Ethics Committee of Wuxi Maternity and Child Health Hospital, China, waived the patient consent form due to the nature of the retrospective study.

### Study participants

This retrospective study was performed in a single university hospital and included 868 women who were diagnosed with GDM at our hospital from January 2022 to December 2022 and were followed up for at least 6 weeks after delivery, from a total of 2175. Pregnant women with a history of diabetes, including GDM in a previous pregnancy, hypertension, and autoimmune diseases, were excluded from this study. Insulin used in this study included NovoRapid or Levemir, or a combination of these two insulin, and the average daily dose of insulin was 20 units (ranging from 4 to 55 units) based on body weight.

GDM was defined as circulating blood glucose levels greater than 5.1 mmol/L at 0 h of 75 g-OGTT (fasting glucose levels), and/or blood glucose levels greater than 10.0 mmol/L at 1 h of 75 g-OGTT, and/or blood glucose levels greater than 8.5 mmol/L at 2 h of 75 g-OGTT, between 24 to 28 weeks of gestational age following the IADPSG guideline [10].

A fasting plasma glucose test was conducted six weeks after delivery (the postpartum visit). Normal postpartum glucose was defined as the fasting plasma glucose levels less than 5.6 mmol/L. Impaired fasting glucose (prediabetes) was defined as the fasting plasma glucose levels between 5.6 to 6.9 mmol/L. Diabetes mellitus is fasting plasma glucose levels greater than 7.0 mmol/L [11]. In our study cohort, only two GDM women had postpartum fasting plasma glucose levels over 7.0 mmol/L. Due to this group's small sample size, we excluded these two cases. Postpartum used in this study was defined as six weeks after delivery.

Data on maternal age at diagnosis, blood glucose levels measured through a 75 g-OGTT at diagnosis, fasting plasma glucose levels measured one day before delivery and six weeks of the postpartum visit, gestational age at diagnosis and delivery, maternal body mass index (BMI) at the time of pregnancy, birthweight, Apgar scores at one and five-minute, complications of pregnancy, weekly weight gain, and treatment options throughout the pregnancy period were collected from the hospital’s electronic database.

Preeclampsia was defined when maternal systolic blood pressure exceeded 140 mmHg and/or maternal diastolic blood pressure exceeded 90 mmHg after 20 weeks of gestational age with impaired liver or renal function and/or a lower platelet count. This definition followed the guidelines of the International Society for the Study of Hypertension in Pregnancy (ISSHP) [12]. Large for gestational age (LGA) was defined as birth weight greater than the 90th percentile for gestational age, following ACOG (The American College of Obstetricians and Gynecologists) guideline [13].

### Statistical analysis

Data from the tables are presented as mean and standard deviation (SD) or as a number and percentage when appropriate. Nonparametric tests (Mann–Whitney test) or Fisher’s exact and odds ratio were performed to assess statistical differences between the groups as suitable. The analysis was conducted using GraphPad Prism software (version 10.1.2.232). A *p* < 0.05 was considered statistically significant.

## Results

The clinical parameters of study participants are listed in Table 1. The mean maternal age was 32 (± 4) years. The mean gestational week at diagnosis was 25^+3^ (± 2^+2^). The mean gestational week at delivery was 38^+3^ (± 1^+2^). The mean BMI at the first prenatal visit was 22.8 (± 3.4) Kg/m^2^.

**Table 1:** Clinical parameters of study participants

Maternal age (years, mean/SD)	32 ± 3.9
Gestational weeks at diagnosis	25^+3^ ± 2^+2^
Gestational weeks at delivery	38^+3^ ± 1^+2^
Parity (n, %)	
1	605 (70%)
2	233 (27%)
≥ 3	28 (3%)
Gravidity (n, %)	
1	415 (48%)
2	245 (28%)
≥ 3	206 (24%)
Birth weight (g, mean/SD)	3254 ± 502
BMI at first prenatal visit (Kg/m^2^, mean/SD)	22.8 ± 3.4

Of these 866 cases, 757 (87%) GDM women presented with normal fasting glucose levels at six weeks of the postpartum visit, while 109 (12.5%) GDM women presented with abnormal postpartum fasting glucose levels (Table 2). Of GDM women with abnormal postpartum glucose levels (n = 109), 63 (57%) women presented with abnormal fasting glucose levels at diagnosis of GDM, which was significantly higher than that in GDM women with normal postpartum fasting glucose levels (342, 45%, p = 0.0179). The odds ratio of GDM women with abnormal fasting glucose levels at diagnosis of GDM presenting with abnormal postpartum fasting glucose levels was 1.662 (95% CI: 1.12, 2.479, p < 0.001) compared to those with normal postpartum fasting glucose levels (Table 2).

**Table 2:** The association of fasting glucose levels at diagnosis with abnormal postpartum fasting glucose levels in GDM women

Fasting glucose at 6 weeks of postpartum	Abnormal fasting glucose at diagnosis (n, %)	Normal fasting glucose at diagnosis (n, %)	Odds ratio (95% CI)
< 5.6 mmol/L (n = 757)	342 (45%)	415 (55%)	1.662 (1.112, 2.479), p = 0.0179,
5.6 – 6.9 mmol/L (n = 109)	63 (57%)	46 (43%)	

Of GDM women with abnormal postpartum glucose levels (n = 109), 60 (55%) presented with abnormal 1-h glucose levels at diagnosis of GDM (Table 3). Of GDM women with normal postpartum glucose levels (n = 757), 357 (47%) presented with abnormal glucose levels of 1-h OGTT at diagnosis of GDM. There was no statistical difference between the two groups (Table 3, p = 0.1775).

**Table 3:** The association of 1-h OGTT at diagnosis with abnormal postpartum fasting glucose levels in GDM women

Fasting glucose at 6 weeks of postpartum	Abnormal 1-h OGTT at diagnosis (n, %)	Normal 1-h OGTT at diagnosis (n, %)	P value
< 5.6 mmol/L (n = 757)	357 (47%)	400 (53%)	0.1775
5.6–6.9 mmol/L (n = 109)	60 (55%)	49 (45%)	

Of GDM women with abnormal postpartum glucose levels (n = 109),70 (64%) presented with abnormal fasting glucose levels one day before the delivery, which was significantly higher than that in GDM women with normal postpartum fasting glucose levels (245, 32%, p < 0.0001, Table 4). The odds ratio of GDM women with abnormal fasting glucose levels one day before delivery presenting with abnormal postpartum fasting glucose levels was 3.751 (95% CI: 2.462, 5.664, p < 0.001) compared to those with normal postpartum fasting glucose levels (Table 4).

**Table 4:** The association of fasting glucose levels one day before delivery with abnormal postpartum fasting glucose levels in GDM women

Fasting glucose at 6 weeks of postpartum	Abnormal fasting glucose one day before delivery (n, %)	Normal fasting glucose one day before delivery (n, %)	Odds ratio (95% CI)
< 5.6 mmol/L (n = 757)	245 (32%)	512 (68%)	3.751 (2.462, 5.664), p < 0.0001)
5.6–6.9 mmol/L (n = 109)	70 (64%)	39 (36%)	

There was no difference in weekly weight gain and BMI at the first prenatal visit between GDM women with and without abnormal postpartum fasting glucose levels (Table 5, p = 0.121, or p = 0.159). However, a significantly higher proportion of GDM women who received additional insulin treatment during pregnancy was seen in women with abnormal postpartum fasting glucose levels compared to those with normal postpartum fasting glucose levels (9% vs 3.5%, p = 0.0048). The odds ratio of GDM women with insulin treatment having abnormal postpartum fasting glucose levels was 3.035 (95% CI: 1.431, 6.074) compared to those with normal postpartum fasting glucose levels. Furthermore, a significantly higher proportion of GDM women who delivered an LGA infant was seen in women with abnormal postpartum fasting glucose levels compared to those with normal postpartum fasting glucose levels (Table 5, 11% VS 5.2%). The odds ratio of GDM women with a delivery of an LGA infant presenting with abnormal postpartum fasting glucose levels was 2.427 (95% CI: 1.233, 4.680, p = 0.016) compared to those with normal postpartum fasting glucose levels. There was no difference in GDM women who developed preeclampsia during pregnancy between women with and without abnormal postpartum fasting glucose levels.

**Table 5:** The association of clinical parameters with abnormal postpartum glucose levels in GDM women

Fasting glucose levels at 6 weeks of postpartum	Additional insulin treatment (n, %)	BMI at the first visit (Kg/m^2^, mean/SD)	Weekly weight gain (kg, mean/SD)	Incidence of LGA (n, %)
< 5.6 mmol/L (n = 757)	N = 27 (3.5%)	22.8 ± 3.35	0.3605 ± 0.2	40 (5.2%)
5.6 – 6.9 mmol/L (n = 109)	N = 10 (9%)	23.4 ± 3.9	0.3936 ± 0.2	12 (11%)
P value	Odds ratio (95% CI) 3.035 (1.431, 6.074, p = 0.0048)	P = 0.159	P = 0.121	Odds ratio (95% CI) 2.427 (1.233, 4.680, p = 0.016)

## Discussion

In this observational study with a relatively large sample size, we found that 12.5% of GDM women developed abnormal postpartum fasting glucose levels (prediabetes level). Abnormal fasting glucose levels at either diagnosis of GDM or one day before delivery were significantly associated with abnormal postpartum fasting glucose levels (six weeks after delivery). Furthermore, GDM women who required additional insulin treatment or delivered an LGA infant showed an increased risk of developing abnormal postpartum fasting glucose levels.

Women with a history of GDM significantly increase the risk of developing type 2 diabetes mellitus later in life. While for most women who had GDM, the glucose levels usually are back to normal range after delivery, more than 25% of GDM women go on to develop type 2 diabetes within three months of postpartum, and 60% of women with a history of GDM develop diabetes within 12 months of postpartum [14]. Consequently, international diabetes communities recommend postpartum glucose monitoring between 6 to 12 weeks of postpartum to detect any glucose abnormalities early and to initiate interventions to prevent or delay the onset of diabetes [15].

Several factors contribute to developing diabetes later in women with a history of GDM. Certain ethnic groups, such as Hispanic, African American, and South Asian, have a 3 to 4 times higher incidence of developing diabetes after GDM compared to Caucasian [16]. That study reported a lower incidence (5.5%) of developing type 2 diabetes after eight years of delivery in East and Central Asians who lived in the United States. Our current study found that only 2 GDM women (0.2%) developed diabetes (postpartum fasting glucose levels over 7.0 mmol/L), and 12.5% of GDM women developed prediabetes monitored on day 42 of postpartum. The relatively lower incidence of diabetes or prediabetes could be due to the diet after giving birth in China. There is a special postpartum diet, which is relatively healthy according to Chinese culture. The traditional postpartum diet includes rich fruits, vegetables, soy products, fish, and limited meats. High intake of fruits, vegetables, and soy products is associated with lower fasting glucose levels in women with a history of GDM [17].

GDM is internationally defined by one or more values exceeding certain thresholds in a 75 g-OGTT: fasting above 5.1 mmol/L, and/or 1 h above 10.0 mmol/L, and/or 2 h above 8.5 mmol/L [10]. However, the interpretation between the fasting glucose levels and 1 h or 2 h OGTT glucose levels could differ. The 1 h or 2 h OGTT measures the body’s response to glucose control, including the glucose clearance from the bloodstream. Whether these three-time points are associated with abnormal postpartum fasting glucose levels remains unclear. Our current study found that GDM women with abnormal fasting glucose levels, but not glucose levels at 1 h OGTT at diagnosis of GDM, significantly increased the risk of developing abnormal postpartum fasting glucose levels. Our data may suggest the difference in response to glucose control among the testing time points.

Additionally, our current study found that the abnormal fasting glucose levels at one day before delivery were strongly associated with the development of abnormal postpartum fasting glucose levels. Uncontrolled glucose levels could indicate the severity of GDM. Furthermore, in our current study, we found that GDM women who required additional insulin treatment were at high risk (threefold) of developing abnormal postpartum fasting glucose levels. Taken together, our data showed that the severity of GDM at diagnosis and GDM women requiring additional insulin treatment during pregnancy were at higher risks for developing abnormal postpartum fasting glucose levels.

GDM is associated with an increased risk of delivery of an LGA infant (reviewed in [18]). While being born an LGA infant does not mean developing insulin resistance or higher plasma glucose levels. However, it does increase the likelihood of abnormal metabolic syndrome in GDM with a history of LGA birth. In our current study, we found a significantly higher risk (2.5-fold) of developing abnormal postpartum fasting glucose levels in GDM women who delivered an LGA infant.

Although BMI and the time of diagnosis of GDM are associated with the risk of abnormal postpartum glucose metabolism, in our current study, we did not see the association of BMI, weekly weight gain, and weeks of GDM diagnosis with abnormal postpartum fasting glucose levels. This could be the lower proportion of obese women in China than in other ethnicities.

There are some limitations in our study. We do not have data on physical activity in these GDM women after delivery, as the lack of physical activity or sedentary lifestyle is associated with an increased risk of postpartum glucose abnormalities. In addition, we also do not have data on breastfeeding in these GDM women. It is well-known that breastfeeding has a protective effect against postpartum glucose abnormalities. Finally, we only compared the fasting glucose on day 42 of postpartum. Future studies are required to analyze the association of fasting glucose levels at diagnosis of GDM and OGTT on day 42 of postpartum.

In conclusion, our study found that the fasting glucose levels at the time of diagnosis of GDM or shortly before delivery are associated with postpartum fasting glucose abnormalities. Our data suggests that fasting glucose levels at diagnosis of GDM could be predictive 

## Data Availability

The datasets used and analyzed during the current study are available from the corresponding author upon reasonable request.
